# Am I Really Bipolar? Personal Accounts of the Experience of Being Diagnosed With Bipolar II Disorder

**DOI:** 10.3389/fpsyg.2020.482715

**Published:** 2020-09-30

**Authors:** Cecilia Johansson, Andrzej Werbart

**Affiliations:** Department of Psychology, Stockholm University, Stockholm, Sweden

**Keywords:** bipolar disorder, diagnosis, early symptoms, depressive episodes, manic episodes, personal accounts, inductive thematic analysis, interviews

## Abstract

Bipolar disorder (BD) is a complex and chronic mental illness with highs and lows beyond the ordinary, which induces a significant risk of suicide. The aim of this study was to explore the experience of being diagnosed with BD and the impact that receiving a correct diagnosis had had on life situations and relationships with others. Semi-structured qualitative interviews were conducted with seven people diagnosed with BD. The results showed that the primary treatment all participants had received or were currently receiving was pharmacotherapy, typically without any psychological component. A major concern that arose was delayed diagnosis, leading to inadequate treatment, and lack of knowledge among professionals about non-typical forms of BD. Moreover, the experiences of others’ reactions were multifold, though generally surprisingly positive. Generally, the participants had learned to recognize, understand and tackle early symptoms of both hypomanic and depressive episodes to avoid developing a full-blown acute episode. This study highlights the crucial importance of a collaborative relationship between the clinician and the patient.

## Introduction

Bipolar disorder (BD) refers to a group of affective disorders, also called mood disorders, which are characterized by depressive episodes and hypomanic or manic episodes (Phillips and Kupfer, 2013). This categorization was introduced by Emil Kraepelin nearly 100 years ago. The term bipolar disorder, however, was first used in 1957, by the German psychiatrist Karl Leonhard (1957) for disorders with both manic and depressive episodes (Leonhard, 1999). However, it took another 20 years for the Diagnostic and Statistical Manual of Mental Disorders (DSM) to replace the initial term ‘manic depression’ with this more modern term when the third edition of the manual was introduced (DSM-III; American Psychiatric Association, 1980).

The fourth edition of the manual (DSM-IV; American Psychiatric Association, 1994) divided the affective disorders into different types and subtypes. The American Psychiatric Association (2019) explains that the aim when developing the DSM-IV was to establish an empirical basis for making the modifications. The fifth edition specified the symptoms further (American Psychiatric Association, 2013). Currently, the bipolar spectrum diagnosis consists of two major types (BD-I and BD-II). BD-I comprises manic episodes followed by depressive episodes. BD-II includes milder forms of mania, so-called hypomanic episodes (Craddock and Jones, 1999), and depressive episodes. In other words, the types differ in how severe the mania typically is. Sometimes BD-I includes psychosis and/or hallucinations, thus being somewhat similar to schizophrenia. According to Craddock and Jones (1999), there are a large number of people who have illnesses with features of both schizophrenia and BD, called schizoaffective disorders. Grande et al. (2016) argued that BD-I might seem to have a more severe symptomatology and prognosis than BD-II due to comorbid symptom severity; however, BD-II has a higher frequency of episodes as well as higher rates of comorbid psychiatric conditions and suicidal behaviors, thus severely impairing the quality of life of persons diagnosed with it.

The DSM-III (American Psychiatric Association, 1980) introduced a third type, ‘cyclothymic disorder,’ referring to conditions similar to BD-II, but which do not qualify for a diagnosis of hypomania or depressive episode. The criteria for cyclothymic disorder are to have had several episodes with symptoms of both hypomania and depression within a 2-year period (1 year for children). Furthermore, the DSM-5 (American Psychiatric Association, 2013) includes ‘other specified bipolar and related disorders’ (an episode shorter than four continuous days), and ‘bipolar disorder not otherwise specified’ (NOS).

BD is a chronic and often devastating illness, easily undiagnosed or misdiagnosed (Singh and Rajput, 2006; Leahy, 2007) because of its complex and diverse nature. However, appropriately diagnosed, it can be effectively treated with a combination of psychological and pharmacological treatment (Leahy, 2007). Successful treatment is crucial, as the suicide rate for those with BD is 20 times higher than for the general population (Ösby et al., 2001; Grande et al., 2016). Moreover, up to 50% of BD patients attempt suicide at least once in their lives and approximately 15–20% eventually die by suicide (Craddock and Jones, 1999; Grande et al., 2016). According to Leahy (2007), BD affects 3–5% of the population. The number is highly dependent on which population the research covers, as well as on which symptoms are included (cf. Angst, 1998; Judd and Akiskal, 2003; Benazzi, 2007). A more recent survey of 11 countries in the Americas, Europe, and Asia (Merikangas et al., 2011) reported an overall lifetime prevalence of bipolar spectrum disorders of 2.4%. Craddock and Jones (1999) claimed that subjects can develop their first BD episode at any time in their lives. However, most commonly, the onset of BD is in late adolescence (Goodwin and Jamison, 1990) or young adulthood (Grande et al., 2016; Rowland and Marwaha, 2018).

The causes of BD are multifaceted and not clear-cut. Clinicians have always known that the illness runs in families (Craddock and Jones, 1999). Some researchers refer to it as a brain disorder, as brain imaging studies of people with BD have shown that the brains of these people may differ from those of healthy individuals (Koch, 2010).

However, brain differences are not necessarily associated with genetic differences, and genetics is not the only leading cause of some people developing BD. Studies of identical twins, who thus share all the same genes, have indicated that other factors as well as genes play a role in the development of BD (Koch, 2010). Researchers have found that both twins do not always develop BD; yet if one of the twins becomes bipolar, then the other is more prone to developing the illness as well (Koch, 2010).

Nevertheless, the importance of genetics in the likelihood of developing BD cannot be neglected. Though no single gene that increases the susceptibility to developing BD has been found, molecular genetics is becoming increasingly advanced, and new tools can be used to find specific genes that are linked to the development of the illness (Craddock and Jones, 1999). Illustrating this very complex nature of the illness, Jamison (1995) now a clinical psychologist, and bipolar herself, states in her book *An Unquiet Mind* (1995, p. 6):

Manic depression distorts moods and thoughts, incites dreadful behaviors, destroys the basis of rational thought, and too often erodes the desire and will to live. It is an illness that is biological in its origins, yet one that feels psychological in the experience of it; an illness that is unique in conferring advantage and pleasure, yet one that brings in its wake almost unendurable suffering and, not infrequently, suicide.

There is a wide array of information about the clinical manuals and diagnostic techniques used to determine whether BD is the explanation for what some people battle every day, such as the ICD-10 classification of mental and behavioral disorders (World Health Organization, 1992), the DSM-5 (American Psychiatric Association, 2013) and the World Health Organization’s Composite International Diagnostic Interview (World Health Organization, 1990). These tools have been both criticized and updated to allow physicians to diagnose those who suffer from BD more easily and more accurately (see Miller et al., 2009; Phillips and Kupfer, 2013).

However, despite the existence of so many different official manuals designed and used to diagnose BD, people suffering from any of the mood disorders usually either go undiagnosed or are misdiagnosed for a prolonged period (Culpepper, 2014). According to a survey conducted by the National Depressive and Manic Depressive Association in the United States, 69% of patients with BD are misdiagnosed initially and over a third are still misdiagnosed 10 years or more after first seeking professional help for their BD symptoms (Lish et al., 1994). The reasons include mistakes in history-taking and limitations in diagnostic criteria, as well as the existence of other health issues that complicate the process of diagnosis (Singh and Rajput, 2006). Two studies found that almost 40% of cases of BD were initially diagnosed with unipolar depression (Ghaemi et al., 1999, 2000). Leahy (2007) asserts that it is too easy to mistake BD for unipolar depression, partly because “few patients voluntarily present to a therapist complaining about manic symptoms such as grandiosity and hypersexuality” (p. 419). According to this author, clinicians should recognize hypomania and mixed states as well as the more typical manic and depressive states. This may be “the single most important event, prior to medication, in the treatment of bipolar patients” (Leahy, 2007, p. 419).

The most common misdiagnosis is unipolar depression (Bowden, 2005; Leahy, 2007). Being diagnosed correctly initially is crucially important for patients who suffer from BD, as misdiagnosis might lead to inappropriate treatment with antidepressants, which in turn can result in manic episodes and trigger long-lasting rapid-cycling BD (Singh and Rajput, 2006). One study of BD patients previously diagnosed with unipolar depression reported that over half of them became manic and one quarter developed rapid-cycling episodes (Altshuler et al., 1995). As reported by Grande et al. (2016), the mean delay between initial help-seeking and correct BD diagnosis is five to 10 years. Furthermore, only 20% of persons with BD are correctly diagnosed within 1 year of seeking help for a depressive episode.

BD is a chronic and severe illness with recurring episodes of depression and mania or hypomania. There is no cure for the disorder, but there are treatments that can reduce the frequency, duration and severity of the episodes (Leahy, 2007). The treatments that should be offered are pharmacological treatments in combination with psychological treatments. However, the most common short- and long-term treatment for BD is medication (Sachs et al., 2000; Culpepper, 2014; Yatham et al., 2018). According to Canadian guidelines, lithium, quetiapine, divalproex, asenapine, aripiprazole, paliperidone, risperidone, and cariprazine, alone or in combination are recommended as first-line treatments for acute mania in BD-I (Yatham et al., 2018). Recommendations for treatment of BD-I acute depression include quetiapine, lurasidone plus lithium or divalproex, and lamotrigine (Yatham et al., 2018). The use of antidepressants must be restricted to episodes of depression, when they should be given alongside a mood stabilizer. Canadian guidelines also recommend monotherapy with a mood stabilizer in the maintenance treatment of BD-II (Yatham et al., 2018).

Few qualitative studies have been conducted on patients’ and their families’ views and experiences of treatment for BD. Lewis (2005) published a short review of unmet needs in the treatment of BD. Seal et al. (2008) conducted a qualitative study of non-treatment-seeking people with hypomanic experiences. Murray et al. (2011) found that people coping well with their BD use strategies alongside effective psychosocial interventions. Fisher et al. (2018) reported having conducted the first in-depth study of patient perspectives on their treatment for BD.

Even though other qualitative studies might not have focused explicitly on the specific topic of receiving a diagnosis, several studies have explored patients’ beliefs about the causes of their problems and about adequate cures. For example, a study of patients with long-standing primary care contact and diffuse somatic problems (Werbart and Levander, 2000) found that the patients had clear and reasonable psychological explanations for their problems, whereas their doctors continued to seek a correct somatic diagnosis. A prospective study of first-episode psychotic patients and their clinicians (Werbart and Levander, 2005) showed that both the patients and the clinicians tended to describe the patients’ problems in a similar way, whereas their beliefs about the causes and cures often diverged. A study of young adults’ ideas of cure prior to psychoanalytic psychotherapy (Philips et al., 2007) found an approaching–distancing dimension, ranging from “processing and understanding” to “avoiding or placing the solution onto others.” An exploratory study of adolescent patients’ causal beliefs regarding depression (Midgley et al., 2017) identified three themes: feeling bewildered about why they were depressed, attributing their difficulties to rejection, victimization, and stress, and blaming themselves for being depressed.

The objective of the present study was to explore patients’ subjective experiences of being diagnosed with BD. How did they experience the process of receiving the diagnosis? What are the positives and negative aspects of getting a diagnosis? How did the patients’ experiences of symptoms and their life situation change through the diagnosis? Finally, what reactions did they receive from others?

## Materials and Methods

### Procedure

To meet the objective of the study, purposive sampling and a self-selected sample of participants interested in sharing their experiences with the researchers was the method of choice. To recruit participants to interview, we posted a brief request in two Facebook groups for people with BD. Those who showed interest in participating in the study received a more detailed information letter. The procedure was repeated 2 weeks later, with the goal of conducting at least six interviews by the end of that month. The only criteria for participation, apart from having been diagnosed with any type of BD, were to be over 18 years of age and to be able to be interviewed face-to-face in Stockholm.

### Participants

Seven participants were interviewed, six of them women. Their ages ranged from 23 to 50 years (*M* = 33; *Md* = 31; *SD* = 9.75). All participants lived in the Stockholm area of Sweden. Five of them had been born in Sweden, one in another Nordic country and one in another European country (both had lived in Sweden for several years and spoke Swedish fluently). At the time of the interviews, three participants were primarily studying, three were on sick leave (although one of them was taking a couple of classes), and another was working as a consultant and designer.

All participants but one had been diagnosed with BD type II; one had a BD-NOS diagnosis in her records, though she described her type as ultra-rapid cycling, which was closer to BD-I. Another participant reported after the interview that, after further evaluations, she had been diagnosed with BD-I. The information about the diagnoses and prescribed medication was provided solely by the participants, and no medical records were accessed.

### Interviews

The semi-structured interviews were conducted by the first author. The first interview was conducted in the participant’s home and the six following interviews took place at Stockholm University. The opening question was “tell me a little about yourself” which was asked mainly to break the ice and create common ground, following Braun and Clarke’s guidelines (2013). This was followed by some more specific questions, indicating thematic areas to be developed by the respondent, with the main focus on the process of being diagnosed with BD:

1. When did your symptoms arise, and how did it affect your life?
2. How did you cope with your symptoms prior to being diagnosed with BD? (Some kind of self-medication?)
3. How and when were you diagnosed with BD? (How did you come into contact with health care services? Your experiences of health care, both during the process of being diagnosed and afterward? Your experiences of medication and other treatments?)
4. How do you feel the diagnosis has had an impact on your social life, family, work, and your view of yourself and life in general?

To obtain more detailed accounts of the participants’ symptoms and their idiosyncratic presentations of the illness, the interviewer asked them to give concrete examples. Interestingly, all the participants spontaneously brought up all the themes addressed in the interview guide. Additionally, while a pre-set interview guide was used, the interviewer also carefully listened to and followed the participants’ stories by, for example, asking more about something they brought up, which follows the hermeneutic practice of interviewing, with participants and interviewers engaging in a dialog which “evolves through questions and responses” (Roulston and Choi, 2018, p. 235). Thus, the participants shape the progression of the interview, as “the researchers sequence questions to generate free-ranging conversations about research topics that are directed by what participants have to say” (p. 233). The interviews lasted about 1 h and were transcribed verbatim. All quotations in this report have been translated into English.

### Data Analysis

The interview transcripts were analyzed by applying inductive, experiential thematic analysis (TA; Braun and Clarke, 2006). Experiential thematic analysis is concerned with how people experience and make sense of their life world. Our use of TA methodology was inductive, as it was grounded in the data and not shaped by pre-existing hypotheses or theories. Moreover, it was explanatory as it involved the researchers’ interpretative activity. One of this method’s strengths is that it can summarize key features based on a large data set, while still offering an in-depth description of what has been found. TA also makes it possible to highlight similarities and differences across the data set.

The qualitative data analysis followed the standardized step-by-step procedure of TA, as prescribed by Braun and Clarke (2006, 2013): (1) familiarization with the narratives and taking notes of relevant items, (2) generating preliminary codes linked, from the point of view of content, to relevant utterances, (3) searching for recurrent themes, i.e., topics, ideas and patterns of meaning that came up repeatedly (4) reviewing and comparing themes and subthemes, (5) defining and labeling themes, (6) describing themes and choosing the most relevant illustrative quotations. TA was conducted by the interviewer (the first author) and audited by the second author.

During the transcription of the interviews, preliminary themes had already begun to emerge, i.e., features that commonly appeared across the data were detected and summarized alongside the transcription. While transcribing each interview, notes were taken, highlighting and marking characteristics that recurred throughout each interview and across the interviews, as well as how many participants had brought up the same topics or subtopics. Though Braun and Clarke (2006, 2013) recommend using computer software, the coding and categorizing of data was in this case conducted manually. Webb (1999) has referred to the familiarization with the data and the intuition that eventually emerges in the process of manual coding as “the artistic part”; something that a computer cannot do. Comparing different approaches, Webb concluded that computerized coding was generally a quicker process for large sets of data (30 subjects or more), but that “the intellectual work of actually conceptualizing can only be done by the brain of the researcher” (Webb, 1999, p. 329). This describes exactly the process which took place in the analysis of the data in this project. When most interviews had been transcribed and coded, a first list of themes was produced. These themes were modified and changed several times, constantly moving back and forth between the whole data set, the coded extracts and the presentation of the results.

As an additional validity check, the number of participants contributing to each theme was scrutinized and reported using labels established by Hill et al. (2005). Thematic categories were considered *general* if they were discerned across all cases or in all but one case (6–7), *typical* if they were present in at least more than half of the cases (4–5), and as a *variant* if they were found in at least two and up to half of the cases (2–3).

The researchers strived for “reflexivity” (Malterud, 2001; Finlay, 2003; Bott, 2010), i.e., the hermeneutic revisiting of data and the evolving comprehension of it, paying attention to the influence of the researcher (personal background, position, preconceptions, and values influencing the investigation). This involved “bracketing” theoretical knowledge and presumptions and holding the emerging insight in abeyance, being open to how each new finding might change an earlier understanding (cf. Fischer, 2009). Before the interviews were conducted, and during the process of developing the interview guide, the first author wrote down and elaborated on presumptions of BD, in line with Braun and Clarke’s (2006) suggestions. This helped the author to become aware of these ideas, so that they could be “bracketed” at the time of the interviews. Additionally, contrary to other scientific practice, the first author did not read through more literature than necessary prior to conducting the study, so that she could engage in the interviews as an ‘empty slate’ which also minimized preconceived ideas on what might appear in the data and likewise the results.

### Researchers

Interviews with the participants and data analysis were conducted by the first author, at the time of the study a psychology student (extended level). The second author, a senior psychoanalyst and professor of psychology, experienced in conducting psychotherapy research and with a special interest in the patients’ perspectives, was involved in the planning of this study, preparing the interview protocol, and conducting audits of the data analysis.

### Member Checking

After the first full draft was finished, all participants were asked to read it and provide their feedback, focusing on two questions: “Did I miss something?” “Is there anything that I have misunderstood?” Six of the participants had nothing to add, approved the draft, and generally thought that the report summarized well their experiences, the theoretical frameworks and the broader picture of BD. One participant asked us to modify some minor details in two examples and approved the revised version.

## Results

The thematic analysis yielded nine overarching themes, representing the participants’ experiences of being diagnosed with BD (Table 1). The themes and subthemes are presented below in what can be seen as a logical timeline. Thus, we start with factors related to the diagnosis itself and to the process of being diagnosed. Subsequently, the experiences of the nature of the illness are explored. Finally, we present the participants’ views of the consequences of the diagnosis. Each theme and subtheme is illustrated with at least one personal account (an indirect quote), sometimes followed by a direct quote.

**TABLE 1:** Number of respondents for each theme and subtheme (*N* = 7).

Theme	Frequency	Label
(1) Obscurity and ambiguity of diagnosis	7	General
(1.1) Scarce information about BD	4	Typical
(1.2) It was by coincidence I found out I had been diagnosed with BD	3	Variant
(2) Delayed diagnosis	5	Typical
(3) Never knowing when it will strike again	7	General
(4) Am I really bipolar?	6	General
(4.1) Comparing one’s own state to that of BD-I	3	Variant
(4.2) Ambivalent feelings about the diagnosis	5	Typical
(5) Understanding own states of mind	7	General
(6) Responses to disclosing diagnosis	7	General
(6.1) Positive responses from others	6	General
(6.2) Others’ lack of acceptance of the illness	3	Variant
(6.3) Friends’ and partners’ inability to handle a loved one’s suffering from BD	2	Variant
(7) The many faces of pharmacotherapy	7	General
(7.1) Resisting treatment	4	Typical
(7.2) Side-effects	5	Typical
(7.3) Reducing initial symptoms of episodes	3	Variant
(8) Health care: improved or not?	7	General
(8.1) Positive treatment experiences	4	Typical
(8.2) Negative aspects of treatment	7	General
(9) Hopes and wishes for better health care	7	General

**Number of participants for each theme and subtheme and label following Hill et al. (2005): general: 6–7 cases; typical: 4–5 cases; variant: 2–3 cases*.*

### (1) Obscurity and Ambiguity of Diagnosis

Typically, participants said that they had been briefly informed by their doctors that they had established the diagnosis of BD but were then left without any further information about what it meant to have this diagnosis and thus this illness. As a variant, they were not even informed by their doctor that a diagnosis had been established.

#### (1.1) Scarce Information About BD

Generally, those participants who had been informed about their diagnosis said that they had not been given much information about the disease. Due to this lack of information about the chronic illness, these participants had to look for information elsewhere to understand what they would have to deal with for the rest of their lives. To most of them this meant researching the Internet and/or joining Facebook groups.

One woman described how she had met a psychiatrist in private practice for 2–3 h. After that time, the doctor said to her: “My tentative diagnosis is that you are bipolar.” The woman explained how she was quite appalled after the appointment, thinking: “A diagnosis … that means … that means I won’t get better,” which had been the goal of seeking help. The only information she was left with was that she had been diagnosed with a chronic illness. The “scientific part” of her, she said, had made her read up on everything she could find on BD, though she was still as confused:

I was very, very confused. Because … it’s not like engineering, that *x* becomes y or *z*… That scared me even more, and I felt … I had to talk to myself and convince myself that I don’t understand those kind of things; the doctors understand this, they know this.

When she went back the following week, the psychiatrist prescribed the mood stabilizing medication lamotrigine. The meeting lasted perhaps 20 min, at the end of which she asked her doctor “But what about this [tentative] diagnosis? Who is going to establish it?” However, without her apparent knowledge, the psychiatrist had already finalized her BD-II diagnosis. In other words, this participant had been diagnosed with BD without being informed about it. She did not receive any information about the illness, either. However, the woman was determined to have her questions answered. She explained how she sent all the questions that she wished to have answered – by mail – to her psychiatrist, giving her the chance to answer them at the appointment the following week.

#### (1.2) It Was by Coincidence I Found Out I Had Been Diagnosed With BD

A variant was that the doctor had not informed the patient that a diagnosis had been established. Three participants reported that they did not know that an official screening for BD had been done, and two participants reported that no screening at all had been done.

One participant explained how she had found out by coincidence that she had in fact been given a bipolar diagnosis 2 years after she had first come into contact with psychiatric care. She had then been treated both in Sweden and in another Nordic country: “I was diagnosed 2 years ago, in the spring, in the first ward, but that doctor never told me; nor did my psychologist, and my contact person found it by coincidence a year [after the diagnosis was established].” This participant clarified at the beginning of the interview that she had never been officially screened for BD, yet her medical records stated that she has BD. She was also convinced that it was just BD that she had. According to her, some family members were bipolar, and she knew that it was very likely that she was, as well.

### (2) Delayed Diagnosis

Typically, being adequately diagnosed was a process which for one reason or another had been delayed. One participant said that the timeframe for being diagnosed with BD is roughly 10 years or more. She confirmed that it had taken a good 10 years for her to be diagnosed with BD-II. In retrospect, she thought her first symptoms of BD had appeared at the age of 15. Despite having had the same psychiatrist for 15 years, she had been diagnosed as late as last year. The first time she had brought up BD with her psychiatrist had been after her depression had evolved into a hypomanic episode:

The first time I brought it up was [7 years ago], and what I remember, and I know that … at that point, a depression ended just like that … sort of … exactly xx o’clock … I mean, a depression doesn’t just end like that … and I became happy, which I guess was good in a way, but I became a bit … it didn’t spiral out of control, but I got a little too happy after having been incredibly unhappy. And then he asked me “How long?” “Well, for 24 h,” and he thought that “Oh well, it has to be four continuous days [as per the DSM-5 criteria].”

This participant continued explaining how she had been discussing and negotiating with her psychiatrist for some years after that about whether or not she is bipolar. The reason why it took so long, she emphasized, was that she was not ready to accept the diagnosis. She also explained that it is quite a tricky illness to detect and diagnose and that her psychiatrist had not seen all her various mood states, mainly because most of them had taken place outside of therapy hours. According to herself, her doctor was …

…smart enough and understood that it is utterly meaningless to give me a diagnosis if I don’t accept it, because it doesn’t make a difference. If he had diagnosed me I most likely would have left, and I suppose he didn’t want that.

Another participant said that she must have been in her 40s when she was diagnosed with BD-II. This was 6–7 years after she had first contacted a psychiatrist. A third participant, a young woman whose first symptoms had appeared at the age of 13, was not diagnosed until 9 years later, when her husband insisted she had to have an evaluation carried out. She had been treated at several mental health institutions since her early teens. Doctors had screened her for ADHD, borderline personality disorder and autism. She had none of them. This same process took place once again when she sought help as an adult – the doctors screened her for ADHD, autism, borderline personality disorder, PTSD, social anxiety and everything else in accordance with the DSM-5 guidance. As she did not score high on any of these, they decided to screen her for BD, as that was the only diagnosis left. Though the result was ambiguous on this test as well, the doctors gave her a temporary diagnosis of BD-II. But they were still not sure and said that they would try giving her medication for BD, to see if it worked. They prescribed lamotrigine and, as she responded fairly well to it, she was officially diagnosed with BD-II.

### (3) Never Knowing When It Will Strike Again

Generally, the worst aspect of the illness for participants, aside from the chronic nature of the BD, has been never knowing when they would lapse into another episode: “At the moment I’m feeling okay, but what if it happens again; what if it gets that bad again?” Another participant said: “I need to rest before it strikes again.”

One variant of the unpredictability of BD was that the participants were constantly cautious and looking for signs of whether or not another episode was looming around the corner. This was a strategy the participants reported having learned some years after having been diagnosed with BD. Whereas this concern and learned detection of early signs was positive overall, it was also often exhausting. A young participant explained how she could find it tiresome and tedious always needing to take care that she got enough sleep, needing to go to doctor’s appointments and check-ups, picking up and taking her medication: “My friends are traveling, or staying out late, but I’m sitting at home with my sleeping pills at exactly 11 o’clock, knowing I have to go to bed. That can be extremely difficult.”

Another variant of unpredictability was lack of control. One participant explained how she “by definition” felt defeated and powerless because of her BD: “I have very good qualifications—a great social network, I don’t have any social problems … but it doesn’t help.” This woman described how despite this, she could not work, because she was struggling with BD as well as other chronic illnesses: “I’ve fought so hard, but it has led nowhere.” Because of this she felt completely out of control:

It is by definition a lack of control, both in relation to my own health and what I am capable of, but also to what I can promise a manager … so what I feel right now is that I cannot promise a manager anything. I have been able to do that before, but I can’t anymore.

Another participant described how one of the hardest aspects of the chronic illness was the necessity to include it in all aspects of her life and take it into consideration in all decisions. She explained how she has to prioritize her sleep, especially when she has slept badly for two nights, though she would rather go out and do something fun with her friends: “Always being grown-up enough to make those decisions, somehow you become your own grown-up, your own parent.” She was only 17 or 18 years old when she was diagnosed with BD.

### (4) Am I Really Bipolar?

Generally, the participants said that at some point they have wondered if they have been correctly diagnosed.

#### (4.1) Comparing One’s Own State to That of BD-I

One variant was that the participants could not comprehend or accept that they have a type of BD because they compared themselves to friends or acquaintances who have BD-I and thus have more severe manic episodes, or simply contrasted their own shifting moods with stereotypical beliefs about what it means to be bipolar. One participant said that she did not know what BD was, other than just the stereotypical mania. She elucidated:

I have had manic depressive people in my surroundings and they’ve had completely different symptoms than I have … been more manic, less depressive, and they’ve had psychoses. So I probably thought that … I think I thought that ‘this must be what it’s like … if you’re bipolar.’ I mean … maybe you see things that aren’t there, you hear things that aren’t there, you hallucinate and things like that, then.

Another participant with the non-typical kind of BD – rapid-cycling – struggled a lot with understanding and accepting her BD diagnosis. She explained that she had had one long, strong phase of denial at the beginning. This was partly because she could not recognize herself at all in the “classical type-I.” She explained how her episodes are constantly ongoing, that she never has several months without an episode, and that her episodes are usually mixed: “There is no daily life for me at all, so it was really hard to find similarities in the typical BD, type-I.” This meant that she at first refused to accept that she is in fact bipolar.

#### (4.2) Ambivalent Feelings About the Diagnosis

Because the aforementioned participant refused to accept her diagnosis and illness, she thought she did not need to take lithium or any other mood stabilizers. To her, it was a matter of a personality disorder, which she had been diagnosed with previously:

You can treat that and get free from it; you can get rid of it and recover when you’re old enough. Bipolar disorder would last my entire life … and to me … that was such a huge change. It was so difficult, I just pushed it away.

Typically, the participants emphasized their struggles with accepting their bipolar diagnosis, mostly because of the severe, unpredictable and chronic nature of the illness. One participant repeatedly mentioned how she had been asking “Why do I have to live with this? Why is something wrong with my brain, or why is something wrong with me?!”

The participants also reported how they had been seeking help, and when receiving the diagnosis, they felt as if they had fought for nothing. “The tremendous fight I have fought, it has led nowhere, and I have a very hard time accepting that. It’s completely incomprehensible,” one of the women explained gloomily. Another woman felt that she had had a really hard time accepting the diagnosis particularly because there is no one typical presentation of symptoms:

I don’t know what I thought or felt when I left [the appointment with her psychiatrist]. I felt, which is true … it isn’t easy for me to accept. But it really isn’t an exact science … and it is extremely different how people react on different medication … You have to start somewhere and try things out.

She continued “What am I going to do now? I understand I need to take my medication … but what am I going to do with this diagnosis? I need help to understand how I should handle it.” What was particularly troublesome for this person was that she realized that being diagnosed with BD meant that she would not recover, which she had been aiming for the whole time. “I have to recover. I need to get rid of this, so that … I mean, a depression is not chronic, it could be, but not necessarily.” That, she knew, was not the case with BD. Nevertheless, she was still hoping to find the right cure.

Yet another person recalled how she had completely refused to accept her diagnosis, and thus also refused medication. The first time someone had screened her for a range of different psychiatric diagnoses, they had found that she scored quite high for BD:

I just laughed at her. My reaction was that I just laughed in her face. Maybe partly because of the stigma … It isn’t something you accept just like that. You don’t want to do that with a chronic illness, really. It wasn’t pleasant to find out.

One of the participants explained how she had denied her BD diagnosis, which doctors had given her after she became manic from antidepressants, though no official evaluation had been done. She had previously been diagnosed with borderline personality disorder. Despite her young age of 23, she had been in close contact with psychiatrists for several years:

It was more about helping me to understand than it was for the mental health clinic to give me the right treatment. I denied my bipolar diagnosis for a very, very long time. I didn’t take mood stabilizing medication. I didn’t take them. I thought that … I am not bipolar … so why should I take the medication for it?

This young woman also gloomily described how she sometimes feels when she sees elderly people in the waiting room at the Affective Disorders Unit:

It is quite a scary thing that when I am 80 … am I going to put on some kind of compression stockings and go out shopping until I run out of money? I find it so hard … that it could be possible to put together a whole life, with a family, children and a job. Then, somehow, it’s so easy to just resort back to the diagnosis, to my illness … and then I can stop taking my medication, because I won’t have to separate from my illness. It’s something I recognize … but everything else, I know nothing about.

Eventually, this person accepted her diagnosis; so much so that she said she was in fact “incredibly grateful” for it. She also met a doctor who emphasized that she had to start taking her medication, or she would not get anywhere, that she wouldn’t get better at all. This was the same doctor who screened her for BD and gave her the official BD-NOS diagnosis, subtype rapid-cycling. The participant explained how this screening made it obvious to her that she does in fact have BD. What she found hardest to accept was always needing to take her disorder into consideration.

### (5) Understanding Own States of Mind

The participants generally reported an improved understanding of the illness and of their early symptoms, both hypomanic and depressive episodes, particularly after they were diagnosed. This was the most positive and essential outcome of having been diagnosed correctly with BD for all participants. Generally, lack of sleep was the most important sign to detect and the most important thing to take care of to avoid yet another up or down episode. One participant, struggling with rapid-cycling BD, said that she had developed a greater understanding of herself, because she understood what happened inside her when she reached a certain state. Somehow this helped her to predict her own episodes. She knew that when she starts losing sleep and planning to do 20 things in one day, then she will become hypomanic or manic the next day: “That’s golden, really, when you live in such a … when it goes up and down so quickly … I learned to stand on my own feet. I learned to adjust to the illness.”

One participant described how much she has learned about BD and how to avoid “getting sick again” and modified her lifestyle thereafter. In particular, she recognizes early symptoms of episodes and can prevent a full-blown hypomania. Another participant explained that she has learned not to continue doing certain things, such as going to parties and being overly social. Instead, she must isolate herself when noticing that she has started swinging upward. This was the complete opposite of what she had been advised to do when spiraling down. Then her doctor bluntly encouraged her to go out and do things.

One woman reported that she has become much better at taking care of herself. When becoming hypomanic, she explained, she has still been grounded in reality to at least some extent. After being diagnosed she treasures that part of her that is still down to earth, despite how tiny that part is. For another participant, the most important thing, some years after having been diagnosed, is that he has developed a kind of self-love. He expressed how he accepts and treasures his body in a completely new light. He explained how he now can see a future; something he could not do for several years.

### (6) Responses to Disclosing Diagnosis

All participants had at least one story to tell about someone else’s reaction. Generally, the responses had been surprisingly positive. However, some participants had experienced negative responses from others, while others felt that their families neglected their illness or mental state.

#### (6.1) Positive Responses From Others

Generally, participants said that their managers, co-workers and families had been very supportive and understanding and played a major role in acknowledging when they began to lapse into an episode. One participant said that her closest friends have all become even closer “now, when they understand why certain things happen.” Another reported that all her friends are very supportive and understanding: “If I cancel something, none of my friends would get mad. They would just tell me they hoped I would feel better.” This participant also highlighted that her manager had been considerate to the utmost when she explained that she might occasionally need to take some time off, to avoid needing to take sick leave for a month or two.

“It helped them understand as well as me,” one participant said. Another woman reported that her manager and co-workers had been informed about her mental health condition even before she was diagnosed. They had been very supportive, and when she was diagnosed with BD, she thought that they were relieved, as they then knew that “it was something which was written on paper.” Another participant reported that his manager knew about his BD diagnosis, so one day when he did not show up at work, the manager called the psychiatric emergency room (ER), who in turn managed to contact his neighbor. This lady, who lived next to him, and also took care of his dog while he was at work, went to check up on him and found that he had tried to commit suicide.

A variant of the positive responses was being able to detect symptoms of episodes, particularly hypomanic episodes. One participant said that most of her peers noticed straight away when she was becoming hypomanic and/or almost manic. She described how she would start dressing differently, would be significantly happier and would speak faster. Her friends, she said, would then ask about her sleeping patterns, and when they were sure she was going upward, they would take her credit cards and passport, to stop her from booking travel impulsively. If needed, they would help her go to the psychiatric ER and be hospitalized for some time. Another participant reported how one of her closest girlfriends was much more attentive to her early symptoms of hypomania than her family was. The friend would sometimes tell her “you’re about to disappear … I want you to come back to reality.” While this was sometimes frustrating, she also felt it was nice and helpful having someone who understood her behaviors before she did.

#### (6.2) Others’ Lack of Acceptance of the Illness

Another variant was that participants felt that others could not handle them when an episode struck. Some also reported that their family members invalidated the illness and the diagnosis altogether. This neglect and stigma arose in one of these families despite the fact that the father was bipolar himself. Another participant’s mother was working at a psychiatric unit, but her husband—the participant’s father—still did not want to hear anything about the illness. This participant reported that after a serious suicide attempt earlier this year, which caused him to lie in a coma for a week, both his parents were more compassionate and worried about him, calling several times a week to check up on him, despite living in another country.

A third participant described how none of her friends or her family wanted to talk about the illness, or even hear about it. She further reported that she received from her relatives comments such as “You don’t look like you’re suffering”; “You, bipolar? No!”; “You look great, you can’t be struggling!” This, she explained, felt as if they were invalidating the competence of her psychiatrist, a doctor she had been seeing for 15 years, and her own understanding of herself. This made her feel very unsafe, because of the severe nature of the illness. Her psychiatrist is the only person around her who picks up the signals demonstrating that another episode has begun, she said.

#### (6.3) Friends’ and Partners’ Inability to Handle a Loved One’s Suffering From BD

One variant of responses to disclosure about BD was an inability to handle the situation. Three participants said that a friend or a partner had left because of the diagnosis. To one of the participants, this was quite devastating, as he had thought that the girl he had met was the love of his life—though he respected her feelings and appreciated her right to leave, as he did not want her to vanish in the process of trying to support him. The other person, whose new date had no understanding of the illness, instigated the break-up herself, as she felt it very important for her peers to know and understand that she is bipolar and what it implies. A third participant gloomily repeated how an old friend of hers had stopped talking to her soon after she was diagnosed with BD-II: “I could think of no other reason for his disappearance.”

### (7) The Many Faces of Pharmacotherapy

All participants have tried multiple types of medication, both prior to being diagnosed with BD and afterward. Typically, they were given antidepressants before being correctly diagnosed. Another variant was that the participants had been put on mood stabilizers to see if they would work. One woman reported having requested lithium even before the official diagnosis had been decided upon. She explained how in her own mind she was certain that she was, in fact, bipolar. Generally, the participants had tried several mood stabilizers, such as lamotrigine, quetiapine and lithium. Typically, they had first tried quetiapine or lamotrigine and if neither of these had worked, lithium was prescribed.

#### (7.1) Resisting Treatment

Typically, the initial approach to medication had been resistance. The reason, according to the two participants who had refused to take mood stabilizers, was primarily their refusal to accept the diagnosis. Another participant clarified how she was skeptical of medication, as she had not noticed any improvement. Her doctor had left it up to her to decide whether the various drugs she was prescribed were effective or not. As a result, she stopped taking them herself, and in retrospect, she was not sure whether she felt better or worse, but she thought she had been relatively stable during the years that she did not take any. Whether this was due to her life circumstances, which at the time were more stable, or because she was not taking any medication, she could not decide.

#### (7.2) Side Effects

Typically, the participants reported several negative side effects or no effect at all. Quetiapine, in particular, gave the participants severe negative side effects. Two participants reported extreme exhaustion for a prolonged period of time, as well as an unnaturally rapid weight gain. One of the participants reported that he had slept for 18 h a day and was unable to stay awake while at work.

One woman said that she had tried pharmacological treatment, though without any result at all. However, she emphasized the severe effect ineffective medication can have on the brain. Whereas a few participants said that the episodes had a strong negative impact on their brain function, this participant emphasized that incorrect medication is even worse. Another woman recounted how sick she had become from taking lithium, as her digestive system stopped working properly.

#### (7.3) Reducing Initial Symptoms of Episodes

One variant of the function of pharmacotherapy was reducing early symptoms. Two participants reported that an increase in dosage during the initial stage of an episode was highly effective. This process had to take place under controlled circumstances; therefore, these two participants were usually hospitalized to prevent a full-blown episode. One of them said that when she becomes acutely depressed, she has to be hospitalized to be put on antidepressants for a short period of time, such as a day or two. This, she explained, turns her depression into a state of mania, if the treatment continues. For another woman, mania, as part of her rapid-cycling BD, was her major problem. Thus, when she was hospitalized, her dosage of lithium was increased significantly. She was also given sedatives, which would help her sleep enough to prevent the mania from developing further.

### (8) Health Care After Diagnosis: Improved or Not?

Positive treatment experiences were typically reported, whereas negative aspects of treatment were a general theme. Typically, pharmacotherapy was the one and only type of treatment the participants claimed to have received. Along with medication, they generally also had a few check-ups a year with a psychiatrist. In some cases, these check-ups were no longer than 20 min. One woman said that these short meetings did not help her at all, as what typically happened was that her psychiatrist concluded that she looked well and healthy, despite her actual condition.

#### (8.1) Positive Treatment Experiences

One participant had already taken part in multiple forms of treatment; though this was not for her BD, but for her borderline personality disorder. Whereas this participant sometimes felt as if she might need better treatment for her BD, most of the time she was grateful for the help with which she had been provided previously, such as psychoeducation. Another participant explained how she had just started cognitive behavioral therapy (CBT) at a new psychiatric service. Whereas she was currently happy with this treatment, she found it rather strange that she had had to call several services around the city, just to be informed which of them offered CBT. Additionally, one participant was very content with the help she has received thus far, as she has had the same private psychiatrist for 15 years.

#### (8.2) Negative Aspects of Treatment

Generally, the participants gave at least one vivid example, demonstrating several aspects of traditional health care, of interventions which arguably had failed. Lack of knowledge and experience related to BD was typically a major problem. For example, one participant had been spiraling down into a depressive episode. Whereas he was aware of it himself and tried to open up to his psychologist about this, the psychologist did not react appropriately. In fact, she even appeared scared when he talked about how he wished to shoot himself. On another occasion, the nurse he met in the open psychiatric ward did not listen carefully when he described how he was becoming drastically worse and also more suicidal. Shortly thereafter, he tried to commit suicide and was in a coma for a week attached to machines to keep him alive. This was the first time that his treatment team became more aware of the signs that he was becoming worse again, such as stopping shaving and developing an unpleasant body odor. He believed psychologists and other psychiatric personnel did not take him seriously when he talked about committing suicide because he seemed so rational and logic when talking about it, even when calling the psychiatric ER.

One young participant who had requested help for her severe depressive episode had been told: “How do you think your husband feels when he has to take care of two children?” When she asked for help, she was blamed for her condition, and the result, ironically, was that her husband had to deal with it all by himself, as she was not offered any treatment, until a couple of years later when she insisted on it. In line with this, another participant said, “You have to be strong and healthy to be sick.”

One participant, who felt content with her treatment from a private psychiatrist, was very disappointed with the public health services. She described one situation when she had called one of the local psychiatric ERs. Although she had been walking around in circles in a park, feeling a strong urge to self-harm, the person on the phone asked her if she was suicidal, to which she said no. He then told her that, according to her records, she was not bipolar. He made the judgment that she was just mildly depressed, which he described as natural. As a result, her urge to self-harm escalated to acute suicidal thoughts. She began to contemplate which bus to walk out in front of but was unable to do this, as it was winter and snowing outside, and all the buses were moving slowly. When recalling this situation, she insisted that it was completely bizarre and explained that she has never asked for help in an acute situation again, even when becoming suicidal.

### (9) Hopes and Wishes for Better Health Care

Whereas one variant of the participants’ experiences with mental health services had been rather positive, generally they were negative. All participants had hopes and requests for better health care. A typical request was to have regular check-ups with the same specialist. A common problem was that the participants never had a chance to get to know their psychiatrists or psychologists. Instead of seeing a psychotherapist or a psychologist, some of them had a nurse as a contact person, whom they saw relatively regularly.

Only one participant happily received CBT; the other six wished for more professional treatment. It was obvious that they were not adequately informed regarding which services were available, and even less regarding what they could expect as part of a treatment plan for BD. One variant was that the participants were on a waiting list for some kind of information session about BD, or a form of CBT. One woman said that she had not heard anything more about this, despite having been put into a queue for “some kind of treatment” almost 10 years ago. She had not even been informed about what kind of treatment it was.

## Discussion

All participants reported both positive and negative aspects of having been diagnosed with BD. Generally, the negative aspects were mostly related to the illness itself, rather than the diagnosis. In spite of the initial denial and sometimes recurring denials, all participants reported feeling that the diagnosis in itself had served a purpose. The most positive function of being adequately diagnosed was an improved understanding of the illness and of early symptoms of episodes, which in turn could prevent a full-blown acute episode. This contrasts with a previous cross-sectional survey among psychiatric inpatients (Marzanski et al., 2002), which showed that almost half of the participants wished to know what was wrong with them.

The strategies the participants in this study reported as successful in reducing severe episodes were similar to those that Murray et al. (2011) found in their multi-design study on the self-management of high functioning individuals with BD, which combined questionnaires and interviews. Understanding one’s own individual signs of BD through close self-monitoring of one’s moods, activities, warning signs and behavioral patterns was a key component in staying well. Another finding was that learning about BD—either through books or other sources such as the Internet, or therapy sessions—helped significantly in understanding one’s own mood patterns. The participants in that study also shared their knowledge with their families, who as a result also learned to detect signs of shifting moods in their relatives suffering from BD. Connecting with others with BD was also important, as was ‘enacting a plan.’ All these themes, found in Murray et al. (2011), apart from following a crisis plan, also emerged in this study. For example, knowledge was a key to even beginning to understand one’s own illness, and the support from friends, family and co-workers was essential to many of the participants.

Despite this, lack of knowledge and information was shown in this study to be a multifaceted and widespread issue. It was evident that the scarcity of information about one’s condition, rights and the availability of treatments was not the only problem. Typically, the participants felt that professionals lacked knowledge about BD and the various forms and presentations of the illness. Five of the seven participants reported at least one situation within the public mental health sector which demonstrated this immense lack of knowledge of this severe illness. Three of these situations were critical; one almost caused death, and another an acute risk of suicide. The other two participants were overtly neglected when actively asking for treatment. One woman described the disappointment when clinical staff praised her for her admirable life circumstances, concluding that she cannot be very ill. “I should be able to get help, although I am trying to do what I can to stay afloat,” she said, somewhat desperately. Additionally, one participant thought in retrospect how strange it was that nobody, despite the many times she had sought help for depression, had asked her about manic symptoms. It was by coincidence and partly due to his unique competence with BD that a psychiatrist noticed what no other clinician previously had—that she is bipolar. This is in line with Leahy’s (2007) argument that BD often goes undiagnosed or misdiagnosed mainly because signs of mania or hypomania are not visible to professionals. There are two reasons for this: firstly, people rarely seek help when they are manic or hypomanic and/or do not disclose information that would portray manic tendencies (Seal et al., 2008); secondly, as illustrated in the above example, clinicians often fail to ask or know about various signs of BD (Leahy, 2007). In agreement with this, two other participants in our study described their experience of the lack of knowledge among mental health professionals, particularly in terms of non-traditional forms of BD, such as ultra-rapid cycling and mixed episodes. This demonstrates that the criteria in the DSM-5 (American Psychiatric Association, 2013) need to be further revised and updated, so that clinicians can learn to acknowledge that BD does not always and only involve pure depressive and manic episodes, thereby contributing to a reduced time gap between the onset of the illness and the correct diagnosis (cf. Grande et al., 2016).

It has been widely recognized that psychological treatment in conjunction with pharmacological intervention is crucial in the care of BD (Leahy, 2007). There is a need for something other than purely pharmacological treatment, especially because of the high suicide risk among BD patients (Ösby et al., 2001; Leahy, 2007). An adequate diagnosis is the first step toward appropriate treatment and, as a result, a more balanced life and a reduction in suicide risk. Delayed diagnosis and misdiagnosis might lead to inappropriate treatment, which aggravates the severity and chronicity of BD and increases the suicide risk. This might also happen when inappropriate treatment follows a correct diagnosis. Nevertheless, despite the great importance of receiving an adequate diagnosis, delayed diagnosis was a major concern in this study. Five of the seven participants reported that they had only recently been diagnosed, although they could have signs and symptoms of BD for more than a decade. Inappropriate treatment and the consequent risks illustrate the typical experience of the participants in this study. Less than half of the participants reported that they were receiving treatment other than purely medication during the period in which they were interviewed. In all cases but one, this was after many years of trying to “scream for help” and to be taken seriously. Accordingly, in a qualitative study of patient and family experiences of treatment of BD-II (Fisher et al., 2018), participants reported mostly medication-based treatment decisions, whereas they expressed a need for “holistic” treatment approaches. Furthermore, they noted that medication decisions carried inherent uncertainty and were based on “trial and error.” They experienced that the treatment decisions were predominantly led by the clinicians, whereas they felt that the patient should be the key decision-maker.

Perhaps the most salient finding in this study was the many stories of how clinical practitioners were failing to provide adequate treatment. One participant described how the nurse he saw regularly, who had worked in public health services for over 40 years, failed to detect both discreet and obviously alarming signs of acute suicidal states. Another remarkable story was narrated by a woman who had once had a strong urge to self-harm and had been pacing around in a park. She had been on the phone with a clinician at the psychiatric ER who ignored her cry for help, claiming that she was not bipolar, simply because her medical records did not state this. Two other women described how they had been told by professionals that they looked healthy and energetic, even when severely depressed and/or hypomanic. Thus, participants typically reported that, despite their active search for help, they were rarely taken seriously, especially when they were insightful about their condition and high functioning. A previous review of patient perspectives on the management of BD (Lewis, 2005) had indicated the need for improved communication and for involving patients in treatment decisions. This can counteract the risk that psychiatric diagnoses label and disempower people rather than ensuring that resources are allocated appropriately (cf. Callard et al., 2013). As suggested in several previous qualitative studies, the clinicians need to be trained to listen carefully to patients’ beliefs about the causes of their problems and the relevant cures, and to be able to negotiate their divergent ideas (cf. Werbart and Levander, 2000, 2005; Philips et al., 2005; Midgley et al., 2017; Fisher et al., 2018).

### Strengths, Limitations, and Further Directions

This study is, to our knowledge, the first qualitative exploration of patients’ experiences of the process of being diagnosed with BD and what the diagnosis means to them. All participants reported having have been diagnosed with BD type II. However, two of the participants claimed to have rapid-cycling BD and one had mixed episodes; additionally, one participant also had symptoms of BD-I. Arguably, data saturation was reached after four interviews. However, the final three, with participants not fully representative of BD-II, added both detail (depth) and width (breadth) to the results. Moreover, only the first author analyzed the raw data. However, the emerging themes were audited and reviewed by the second author. Furthermore, to enhance the trustworthiness of our results, we applied member checking (participant validation) to obtain participant feedback on our presentation of their experiences. Following the request of one of the participants, we modified some minor details in two examples, not changing the meaning of the quotations. Another participant emphasized that the report brought up the most important aspects of her experiences. Still another participant stressed the lack of knowledge and competence to work with BD patients that was prevalent among clinicians. She also added that for patients, processing the BD diagnosis is crucial, rather than purely being medicated for it. A further limitation is that only one man was interviewed; thus, this report mainly represents women diagnosed with BD-II. On the other hand, BD-II is most common in women (Grande et al., 2016).

The participants were recruited via two Facebook groups for people with BD. All participants were highly motivated to take part in this study and had a strong need to tell their stories. All participants were also encouraged to get in touch after the interview if they wanted to add something or had any questions. The interviewer invited all participants to be part of the process of writing and editing the article. Asked to provide their comments on the “Results” section, the participants highlighted the importance of investigating the subjective experience of being diagnosed with BD and expressed their appreciation that this study was being conducted. We can therefore also assume that being part of the study was a helpful experience to them, as they had the chance to share their stories and to be heard in terms of what they had experienced throughout the process of being diagnosed and receiving treatment.

All participants provided detailed information about their psychiatric diagnoses and medication. All were critical of how they had been treated in the health care system and reported receiving strong support from their friends and families. Being active in Facebook groups, the participants were also deeply committed to understanding and managing their conditions. Thus, their stories might not be representative of the individuals with BD who are not active in online discussion groups and who do not have access to comparable social support. Furthermore, none of the participants was in an acute ongoing episode and all of them seemed to be at a point of control over their condition. We can assume that persons who sign up for a Facebook group might differ in some way from other persons with the same diagnosis who do not seek such support. On the other hand, the privileged position of the participants could contribute to a deeper and not otherwise accessible understanding of the experience of receiving a BD diagnosis. Even if the strength of this study is the focus on the participants’ subjective perspective, all data were based on retrospective recall and an independent confirmation of the anamneses, diagnoses, and treatments is lacking.

Future studies should include more men and a greater variety of BD-spectrum diagnoses, and also look at the role of comorbidity in the process of diagnosing. A larger, cross-cultural study could compare different health care systems. Finally, a prospective longitudinal study could throw more light on the process of diagnosing as well as on the participants’ processing of receiving a diagnosis.

## Conclusion

This study found that those who have been diagnosed with BD often go through several phases of denial. Once they have accepted their diagnosis, they can begin to learn more about the illness, their own distinctive symptoms, and how to handle them. Despite the obvious beneficial effect appropriate treatment has been found to have regarding reduction in severity and chronicity, and on the management of BD symptoms, as well as sustained reduction in health care services usage (Leahy, 2007; Murray et al., 2011; Abbass et al., 2019), this study revealed an alarmingly widespread scarcity of such treatments. Lack of knowledge, both among professionals and the participants, was another main issue, despite the importance of knowing one’s own illness and recognizing its symptoms and signs. Only one participant reported that she had received both CBT and psychoeducation. Nevertheless, the participants typically reported that they had learned about their illness and what different signs imply, though mostly through their own efforts.

Lack of appropriate treatment for BD, as well as lack of knowledge both among the general population and among professionals, were core issues which need to be addressed. As also emphasized by Leahy (2007) and Murray et al. (2011), professionals need more information about what to look for, so that an adequate diagnosis can be established quickly, and individuals with BD can accordingly receive appropriate treatment. Adequate treatment of BD should involve pharmacotherapy, providing information, psychoeducation, help with self-management and the option of psychotherapy (cf. Daggenvoorde et al., 2013). Most importantly, our study highlights the crucial importance of a collaborative relationship between the clinician and the patient, as also stressed by Murray et al. (2011). Mental health professionals need training to listen carefully and with respect to each specific individual, and to bear in mind that an inner battle is far from always visible.

## Data Availability Statement

The datasets generated for this study will not be made publicly available. The participants signed the informed consent form that included the statement that the verbatim transcripts of the interviews will only be available to the researchers conducting the present study.

## Ethics Statement

Ethical review and approval was not required for the study on human participants in accordance with the local legislation and institutional requirements. The patients/participants provided their written informed consent to participate in this study.

## Author Contributions

CJ planned and designed the work in cooperation with AW. CJ was responsible for acquisition of all the data included, analysis and interpretation of the data for the work, and early drafting. AW continuously scrutinized data analysis, interpretation of results, and early drafting, and contributed with critical revision in the later stages of the work. Both authors have given final approval of the version to be published and agreed to be accountable for all aspects of the work.

## Conflict of Interest

The authors declare that the research was conducted in the absence of any commercial or financial relationships that could be construed as a potential conflict of interest.
